# Expression of DNA topoisomerase II-α: Clinical significance in laryngeal carcinoma

**DOI:** 10.3892/ol.2014.2367

**Published:** 2014-07-22

**Authors:** YAN FENG, HAILI ZHANG, WEI GAO, SHUXIN WEN, HUI HUANGFU, RUIFANG SUN, WEI BAI, BINQUAN WANG

**Affiliations:** 1 Department of Otolaryngology, Head and Neck Surgery, The First Hospital, Shanxi Medical University, Taiyuan, Shanxi 030001, P.R. China; 2 Nursing College of Shanxi Medical University, Taiyuan, Shanxi 030001, P.R. China; 3 Department of Pathology, Shanxi Cancer Hospital, Taiyuan, Shanxi 030013, P.R. China

**Keywords:** DNA topoisomerase II-α, immunohistochemistry, chromosome 17 aneuploidy, laryngeal squamous cell carcinoma, fluorescence *in situ* hybridization

## Abstract

DNA topoisomerase II-α (Topo II-α) is essential for numerous cell processes, including DNA replication, transcription, recombination, and chromosome separation and condensation. Altered Topo II-α expression may lead to carcinogenesis and cancer progression. The aim of the present study was to investigate the association between Topo II-α expression levels and clinicopathological data from laryngeal cancer patients. Immunohistochemistry was used to analyze Topo II-α expression in laryngeal squamous cell carcinoma and distant healthy tissues obtained from 70 patients. In addition, fluorescence *in situ* hybridization was used to detect Topo II-α amplification and chromosome 17 ploidy using a laryngeal cancer tissue microarray. The expression of Topo II-α protein was detected in 71.43% (50/70) of laryngeal carcinoma tissues, in contrast to 9% of healthy tissues (2/22). Furthermore, the expression of Topo II-α protein was found to be associated with tumor de-differentiation and advanced tumor T stage. However, the expression of Topo II-α protein was not identified to be associated with *Topo II-α* amplification in laryngeal carcinoma, although was found to positively correlate with chromosome 17 aneuploidy (P<0.05). A higher aneuploidy rate contributed to increased expression levels of Topo II-α protein. Aberrant Topo II-α expression and chromosome 17 aneuploidy contributed to the development and progression of laryngeal cancer, indicating that targeting Topo II-α may provide a treatment strategy for patients with laryngeal cancer.

## Introduction

Laryngeal cancer is a common type of head and neck malignancy. In the United States, there were an estimated 12,260 novel cases of laryngeal cancer in 2013 (1). Tobacco smoking and alcohol consumption are the most significant risk factors for laryngeal cancer, thus, smoking cessation and decreased alcohol intake may reduce the incidence of this cancer (2). The prognosis of patients with laryngeal cancer is closely associated with the tumor size, location, histological grade, patient age and the presence of lymph node or distant metastasis (3). For example, the five-year survival rate of early laryngeal cancer may be as high as 80–90%, whereas the five-year survival rate of advanced laryngeal cancer declines to ~60% (4). Thus, early diagnosis is considered to be critical for improving the survival of patients. Novel approaches to identify biomarkers may facilitate clinicians with the early identification of laryngeal cancer and studies on the biological behavior of this cancer may provide valuable information for clinical treatment.

The DNA topoisomerase II-α (*Topo II-α)* gene, localized at chromosome 17q21–22, encodes a 170-kD protein that regulates the dynamic changes in the spatial structure of nucleic acids (5). Topo II-α is a key enzyme, which maintains the physiological functions of nucleic acids (6) and is important in numerous cellular processes, including DNA replication, recombination, chromosome separation, and condensation and gene transcription (7). At present, the majority of the anticancer agents that have been developed interfere with DNA replication, recombination and gene expression in tumor cells. Thus, Topo II-α is the common target of antitumor drugs, including anthracycline, actinomycin and podophyllotoxin. These agents promote enzyme-mediated DNA cleavage by stabilizing the dissociation-prone complex between Topo II-α and DNA, leading to the accumulation of DNA double-strand breaks and subsequently resulting in tumor cell apoptosis (8). Thus far, Topo II-α amplification and protein expression have been extensively investigated in a variety of cancers, including breast cancer, testicular teratoma, bladder transitional cell carcinoma, meningioma, glioma, liver cancer and endometrial cancer (9–12). Topo II-α expression was found to be associated with tumor invasion and recurrence, as well as with the prognosis of different cancers (13,14). Previous studies have shown that levels of Topo II-α are associated with the responsiveness of tumors to chemotherapy and radiotherapy (15). Thus, in the current study, the expression of Topo II-α protein was analyzed in laryngeal cancer and adjacent tissues using immunohistochemistry, and its association with clinicopathological data was determined. *Topo II-α* amplification was also examined in addition to chromosome 17 aneuploidy using fluorescence *in situ* hybridization with the aim of identifying the mechanisms by which Topo II-α protein expression is regulated in laryngeal cancer.

## Patients and methods

### Patients

A total of 77 patients with pathologically confirmed laryngeal squamous cell carcinoma were enrolled in the present study and the patients underwent surgical tumor resection at Shanxi Cancer Hospital (Taiyuan, China) between January 2005 and December 2007. No patients received radiotherapy or chemotherapy prior to surgery. This study included 71 males and six females with a mean age of 59.3 years (range, 41–79 years). Regarding tumor localization, 40 (51.95%) patients were diagnosed with supraglottic cancer, 30 (3.90%) with glottic cancer, six (7.79%) with subglottic cancer and one (1.30%) patient was diagnosed with an unknown cancer location due to incomplete data. Histologically, five (6.49%) tumors were well-differentiated, 64 (83.12%) were moderately differentiated and eight (10.39%) were poorly differentiated squamous cell carcinomas. Regarding the clinical T stage, four patients (5.2%) were identified with the T1 stage of disease, 26 patients (33.77%) with the T2 stage, 31 patients (40.26%) with the T3 stage, 11 patients (14.29) with the T4 stage and five patients (6.49%) with an unknown T stage due to incomplete information. A total of 15 patients (19.48%) exhibited lymph node metastasis, however, no patients were identified to exhibit distant metastases. A total of 22 pairs of laryngeal cancer and distant healthy tissues were available for the study, whereas in the remaining 55 cases only laryngeal cancer tissues were available. The present study was approved by the Ethics Committee of Shanxi Cancer Hospital and informed consent was obtained from all patients prior to enrollment.

### Immunohistochemistry

A mouse monoclonal anti-human Topo II-α antibody and PV-9000 general-purpose two-step immunohistochemical detection kit were purchased from Beijing Zhongshan Golden Bridge Biotechnology Co., Ltd. (Beijing, China). A 3,3′-diaminobenzidine kit was purchased from Fuzhou Maixin Biotechnology Development Co., Ltd. (Fuzhou, China). For immunohistochemistry,77 laryngeal squamous cell carcinoma tissue sections and 22 distant healthy tissue specimens were deparaffinized, rehydrated and subjected to antigen retrieval using a citrate buffer (Beijing Zhongshan Golden Bridge Biotechnology Co., Ltd.) at high pressure. All procedures were performed according to the manufacturer’s instructions. Phosphate-buffered saline (Beijing Zhongshan Golden Bridge Biotechnology Co., Ltd.) served as the blank control to replace the primary antibody. The presence of Topo II-α protein in the nucleus of positive tumor cells was observed as uniform brown and yellow particles. Three high-power fields were randomly selected from each tissue section and the mean percentage of positive cells was defined as the percentage of positive cells in each tissue section. Percentage (%) of staining of Topo II-α protein was graded as follows (16): (−), <10% positive cells; (+), 10–25% positive cells; (++), 26–75% positive cells; and (+++), 76–100% positive cells. The grades between (+) and (+++) were considered to represent positive Topo II-α expression, while the grades between (−) and (+) were considered to represent low Topo II-α expression and grades between (++) and (+++) as high Topo II-α expression.

### Fluorescence in situ hybridization

Paraffin blocks from 55 laryngeal squamous cell carcinoma tissues and 22 distant healthy tissue specimens were retrieved from the Pathology Department of Shanxi Cancer Hospital and were used to prepare two 5×6 tissue microarrays (2.0 mm) for fluorescence *in situ* hybridization.

The tissue microarray sections were heated at 65°C for 120 min and deparaffinized with xylene, rehydrated in a series of ethanol, and washed in distilled water for 1 min. For fluorescence *in situ* hybridization, the sections were placed in a water bath at 90°C for 30 min and rinsed twice for 5 min in 2X saline-sodium citrate (SSC) solution at room temperature. Next, the sections were denatured and hybridized with a fluorescent-labeled Topo II-α cRNA probe (Beijing Golden Bodhisattva Jia Medical Technology Co., Ltd., Beijing, China) at 37°C overnight in a hybridization oven (ThermoBrite 5500-24; Abbott Laboratories, Abbott Park, IL, USA) according to the manufacturer’s instructions. The sections were submerged in 2X SSC until the coverslip detached naturally. Next, the sections were counterstained with 4′,6-diamidino-2-phenylindole for 10–20 min and were visualized using a laser scanning confocal microscope (Leica SP-5; Leica, Mannheim, Germany). The fluorescent signal of the tissue specimens was reviewed and quantified to determine the DNA content using the copy number of *Topo II-α*. Digoxigenin-labeled human papillomaviruses 16/18-positive tissue sections served as the positive control and glycerol replaced the probes and served as a negative control. In healthy cells, the single interphase nucleus contained two red and two green signals. In tumor cells, particularly those with abnormal *Topo II-α* amplification, the single interphase nucleus contained more than two red signals. The ratio was calculated as follows: Ratio = total number of red signals in 30 nuclei/total number of green signals in 30 nuclei. A ratio of <1.8 indicated no *Topo II-α* gene amplification and a ratio of >2.2 indicated the presence of Topo II-α gene amplification according to previous Her2-neu studies (17). When the ratio was between 1.8 and 2.2, the number of cells counted was increased to 100 to determine the final result. To determine chromosome 17 aneuploidy, the mean number of chromosome 17 in each cell was determined, whereby values between1.76 and 2.25 indicated diploidy, whereas a value ≥2.26 indicated polyploidy (18).

### Statistical analysis

SPSS version 15.0 statistical software (SPSS, Inc., Chicago, IL, USA) was used for all statistical analyses. Comparison between the groups was performed using χ^2^ test or Fisher’s exact test. The association between *Topo II-α* gene expression and other factors was analyzed using Spearman’s test. P<0.05 was considered to indicate a statistically significant difference.

## Results

### Expression of Topo II-α protein in laryngeal carcinoma tissue specimens

**Figure 1:**
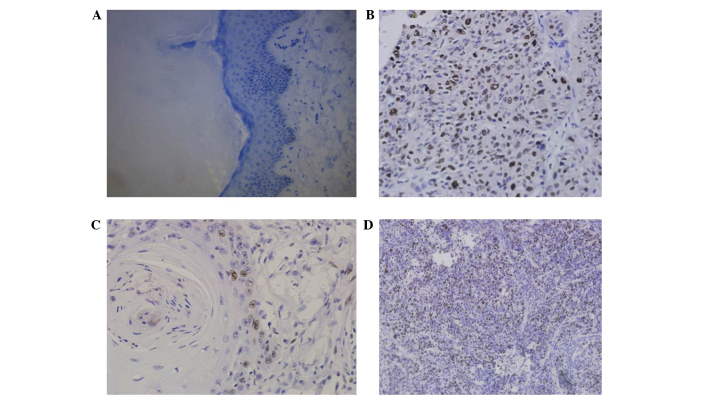
Immunohistochemical detection of Topo II-α protein expression in laryngeal cancer tissue specimens compared with distant healthy tissues. (A) Healthy tissue without Topo II-α expression. (B) Laryngeal cancer tissue with Topo II-α expression (tumor cell nuclei appeared brown and yellow in color). (C) Well-differentiated laryngeal cancer. (D) Poorly differentiated laryngeal cancer. (Magnification: A, ×400; B, ×400; C, ×400; and D, ×200).

During immunohistochemical staining, certain tissue sections samples detached from the glass slides, and thus, only 70 samples were available for data analysis. Topo II-α was observed to be predominantly expressed in the nucleus of the cells, appearing as yellow, or brown and yellow in color (Fig. 1). Topo II-α expression was positive in 71.43% (50/70) laryngeal cancer tissue specimens with a low expression rate of 52.11% (37/70) and high expression rate of 47.14% (33/70; Table I). By contrast, the Topo II-α protein was only expressed in 9% (2/22) of the distant healthy laryngeal tissues (P<0.05).

The association between Topo II-α expression and patient’s clinicopathological data was investigated. It was found that when compared with moderately and poorly differentiated tumors, well-differentiated tumors expressed the Topo II-α protein at a significantly lower level (P<0.05; Table II). Furthermore, when compared with the stage T3 + T4 group, the T1 + T2 tumors also expressed low levels of Topo II-α protein (P<0.05). In tumors without lymph node metastasis, the expression of Topo II-α protein was low, whereas in tumors with lymph node metastasis, the expression of Topo II-α protein was higher (50% [7/14]), however no significant differences were identified (P>0.05; Table II). In addition, 54.05% (20/37) of supraglottic cancer exhibited low Topo II-α expression, whereas 45.95% (17/37) of tumors exhibited high expression. Similarly, 53.85% (14/26) of glottic cancer exhibited low expression levels of Topo II-α protein, whereas 46.15% (12/26) of glottic cancers exhibited high levels of Topo II-α protein expression. By contrast, low and high levels of Topo II-α protein were observed in equal numbers of subglottic cancer tissue samples (50% [3/6]), however, no significant difference was identified between the expression of Topo II-α protein and the different tumor localizations (P>0.05; Table II).

### Chromosome 17 ploidy and its association with the expression of Topo II-α protein in laryngeal carcinoma tissue specimens

**Figure 2:**
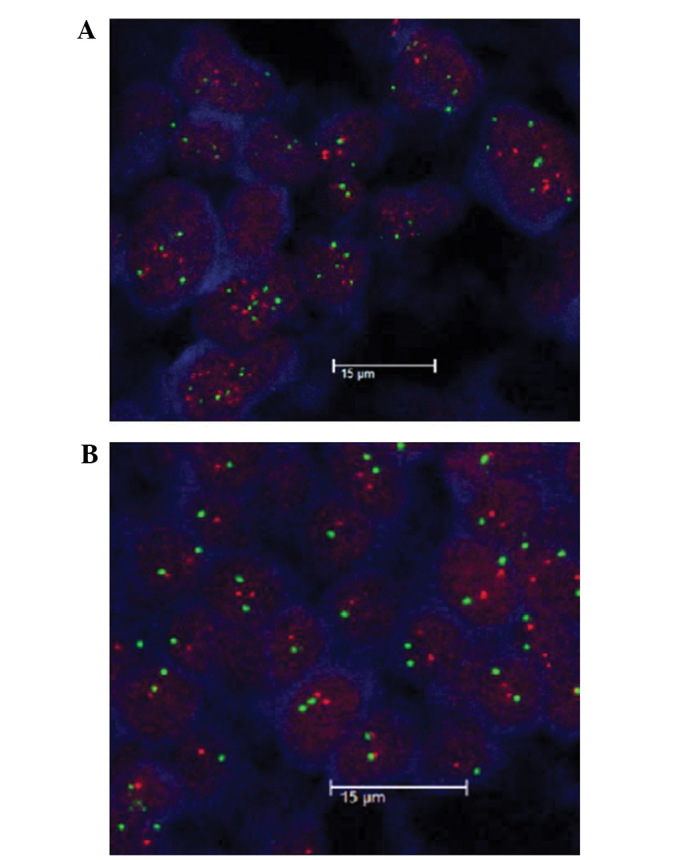
Fluorescence *in situ* hybridization analysis of *Topo II-α* amplification and chromosome 17 polyploid in laryngeal cancer tissue specimens. Blue signals represent the nucleus, green signals represent chromosome 17 and red signals represents *Topo II-α*. (A) *Topo II-α* amplification. *Topo II-α* gene was amplified and chromosome 17 was found to be polyploid (magnification, ×630). (B) Chromosome 17 aneuploidy. *Topo II-α* was not amplified and chromosome 17 was found to be diploid (magnification, ×630).

The overall aneuploidy rate of chromosome 17 was 45.83% (22/48) in the laryngeal carcinoma tissue specimens. Chromosome 17 aneuploidy was also found in 6/27 (22.22%) of cases with (−/+) expression of Topo II-α protein when compared with 16/21 (76.19%) in cases with (++/+++) expression of Topo II-α protein (P<0.05). These results revealed that a higher rate of chromosome 17 aneuploidy is associated with a higher expression of Topo II-α protein (Table III). Chromosome 17 aneuploidy was also found to be associated with the clinicopathological data (Table IV; Fig. 2).

### Topo II-α amplification and association with the expression of Topo II-α protein in laryngeal carcinoma tissue specimens

*Topo II-α* amplification was detected in 7/48 (14.58%) laryngeal carcinoma tissue specimens. The association between *Topo II-α* amplification and the expression of Topo II-α protein was investigated in laryngeal carcinoma tissue specimens. The results showed that Topo II-α protein was not expressed in 15 cases, whereas 12 cases expressed low levels of Topo II-α protein (+), 17 expressed moderate levels (++) and four expressed high levels (+++). With regards to *Topo II-α* amplification, seven cases exhibited amplification, with one case demonstrating negative Topo II-α protein expression, one case demonstrating (+) Topo II-α protein expression, and five cases demonstrating (++) Topo II-α protein expression (P>0.05; Table V). However, *Topo II-α* amplification was not found to be associated with any clinicopathological data from laryngeal cancer patients (data not shown; Fig. 2).

## Discussion

In the present study, the expression of Topo II-α protein, *Topo II-α* amplification and chromosome 17 ploidy were analyzed in laryngeal cancer tissues. In addition, the association between the expression levels of Topo II-α protein and the clinicopathological data from the patients, as well as the association between Topo II-α expression, *Topo II-α* amplification and chromosome 17 ploidy was analyzed. It was found that the expression of Topo II-α protein was upregulated in tumor tissues when compared with their healthy counterparts. Furthermore, the expression of Topo II-α protein was associated with tumor de-differentiation and advanced tumor T stage, although not with clinical classification or cervical lymph node metastasis of laryngeal carcinoma. The levels of Topo II-α protein expression were not found to be associated with *Topo II-α* amplification, however, were closely associated with the aneuploidy of chromosome 17. The findings of the current study indicate that the expression of Topo II-α protein may be used as a tumor marker and inhibition of Topo II-α activity may be further developed as a therapeutic target for laryngeal cancer patients.

At present, few studies have assessed Topo II-α expression and the association between Topo II-α protein and clinicopathological data from laryngeal cancer patients; however, the majority of these studies have reached consistent conclusions. Horibe *et al* (19) performed an immunohistochemical analysis of 63 cases of early glottic laryngeal carcinoma and 10 cases of normal laryngeal mucosa, and showed that the expression of Topo II-α protein was significantly increased in early glottic laryngeal carcinoma tissue when compared with the normal laryngeal mucosa. Furthermore, Shvero *et al* (20) immunohistochemically analyzed Topo II-α protein expression in 50 cases of laryngeal carcinoma, and demonstrated that the expression level of Topo II-α protein was associated with the pathological grade, clinical stage, postoperative survival and recurrence rate, indicating that Topo II-α protein overexpression is associated with a poor prognosis. In addition, Deng *et al* (21) investigated Topo II-α protein expression in 24 cases of laryngeal squamous cell carcinoma and eight cases of vocal cord polyps using immunohistochemistry and found that the positive expression of Topo II-α protein was significantly higher in patients with laryngeal squamous cell carcinoma compared with that in patients with vocal cord polyps and that the levels of Topo II-α expression were associated with the degree of tumor differentiation. Guo (22) also conducted an immunohistochemical study of Topo II-α protein expression in 50 cases of laryngeal cancer and revealed that Topo II-α protein expression correlated with the degree of differentiation, clinical stage and lymph node metastasis, furthermore, poor differentiation, higher clinical stage and the presence of lymph node metastasis was found to be associated with elevated Topo II-α protein expression. Altogether, these studies indicated that the levels of Topo II-α expression may serve to predict tumor de-differentiation or prognosis for patients with laryngeal squamous cell carcinoma. The results of the current study are consistent with these studies, however, the current study did not include survival data.

Furthermore, the potential cause of Topo II-α overexpression was assessed in laryngeal cancer tissue specimens using a fluorescence *in situ* hybridization technique. It was found that only seven out of 48 cases exhibited *Topo II-α* amplification, however, 22 cases exhibited chromosome 17 aneuploidy. In the current study, an association between the expression levels of the Topo II-α protein and *Topo II-α* amplification was not identified, however, chromosome 17 aneuploidy was identified to be associated with the expression levels of the Topo II-α protein. Expression of the Topo II-α protein increased in line with an increase in chromosome 17 aneuploidy, however, expression of Topo II-α protein levels in laryngeal carcinoma was not found to be associated with the Topo II-α mRNA levels for reasons that remain unclear. This indicates that chromosome 17 aneuploidy may induce the stability of the Topo II-α mRNA or protein. In conclusion, the results of the current study indicate that the aberrant expression of the Topo II-α protein may be involved in the development and progression of laryngeal cancer, and that the assessment of Topo II-α protein expression levels may provide insightful information regarding potential targets for use in laryngeal cancer treatment.

## Figures and Tables

**Table I:** Topo II-α expression in 70 laryngeal squamous cell carcinoma tissues.

		Topo II-α expression, n			
		
Tissue	Patients, n	(−)	(+)	(++)	(+++)
Cancer	70	20	17	29	4
Healthy	22	20	2	0	0

**Table II:** Association of Topo II-α expression with clinicopathological parameters of laryngeal squamous cell carcinoma patients.

		Topo II-α expression, n		
Clinicopathological parameter	Patients, n	(−/+)	(++/+++)	P-value
Gender				
Male	69	36	33	>0.05
Female	1	1	0	
Age, years				
<60	36	20	16	>0.05
≥60	34	17	17	
Clinical type				
Supraglottic	37	20	17	>0.05
Glottic	26	14	12	
Subglottic	6	3	3	
Pathological grade				
Well-differentiated	5	4	1	<0.05
Moderately/poorly differentiated	65	33	32	
T stage				
T1 + T2	26	17	9	<0.05
T3 + T4	40	20	20	
N stage				
N0	56	30	26	>0.05
N1–3	14	7	7	

**Table III:** Association between Topo II-α protein expression and chromosome 17 ploidy in 48 laryngeal cancer patients.

Group	Patients, n	Diploidy, n (%)	Polyploidy, n (%)
IHC (−)/FISH (−)	14	12 (85.71)	2 (14.29)
IHC (+)/FISH (−)	11	7 (63.64)	4 (36.36)
IHC (++)/FISH (−)	12	4 (33.33)	8 (66.67)
IHC (+++)/FISH (−)	4	1 (25.00)	3 (75.00)
IHC (−)/FISH (+)	1	1 (100.00)	0
IHC (+)/FISH (+)	1	1 (100.00)	0
IHC (++)/FISH (+)	5	0	5 (100.00)

IHC, immunohistochemistry; FISH, fluorescence *in situ* hybridization.

**Table IV:** Association between chromosome 17 aneuploidy and clinicopathological data from laryngeal cancer patients.

		Chromosome 17 aneuploidy		
			
Clinicopathological parameter	Patients, n	Diploidy	Aneuploidy	P-value
Age, years				
<60	22	15	7	>0.05
≥60	26	11	15	
Gender				
Male	47	26	21	>0.05
Female	1	0	1	
Clinical type				
Supraglottic	25	16	9	>0.05
Glottic	18	8	10	
Subglottic	5	2	3	
Pathological grade				
Well-differentiated	3	2	1	>0.05
Moderately/poorly differentiated	45	24	21	
T stage				
T1 + T2	15	12	3	<0.05
T3 + T4	31	13	18	
N stage				
N0	36	20	16	>0.05
N1–3	12	6	6	

**Table V:** Association between Topo II-α protein expression and *Topo II-α* amplification in 48 patients with laryngeal squamous cell carcinoma.

		FISH	
		
IHC	Patents, n	Non-amplification	Amplification
(−)	15	14	1
(+)	12	11	1
(++)	17	12	5
(+++)	4	4	0

IHC, immunohistochemistry; FISH, fluorescence *in situ* hybridization.
